# Commentary: Early Screening Parameters for Dysphagia in Acute Ischemic Stroke

**DOI:** 10.3389/fneur.2018.00148

**Published:** 2018-03-20

**Authors:** Massimiliano Toscano, Alessandro Viganò, Vittorio Di Piero

**Affiliations:** ^1^Department of Human Neuroscience, Sapienza University of Rome, Rome, Italy

**Keywords:** NIH Stroke Scale, Dysphagia, Stroke

We read with great interest the article by Henke et al. (1) aimed to identify early assessable predictors of dysphagia in the acute phase of ischemic stroke. A multivariate logistic regression analysis revealed higher age, male gender, and higher stroke severity [as assessed by NIH stroke scale (NIHSS)] to be independent predictor of poststroke dysphagia. Moreover, ROC analysis showed that in the acute phase of stroke NIHSS score of 4.5 was the best cut-off between dysphagic and non-dysphagic patients.

This research field certainly has a remarkable clinical interest, because it may lead to individuate those acute stroke patients who may benefit from a more detailed assessment of their swallow function (i.e., using fiberoptic endoscopic evaluation of swallowing or videofluoroscopy). However, an NIHSS cut-off for dysphagia of 4.5, by excluding only stroke patients with a very mild deficit, is probably of limited clinical usefulness. In other words, since the majority of stroke is of moderate-severe degree, it appears to be of little help in detecting only those patients who may benefit from a more detailed assessment of their swallowing function.

In our previous study (2), we prospectively examined consecutive patients with both ischemic and hemorrhagic stroke, by assessing their swallowing function in the acute phase (i.e., within 72 h from stroke onset) and after 14 days as well; patients were then classified as persistent dysphagic, non-persistent dysphagic, or non-dysphagic.

In line with Henke and co-workers, stroke severity was an independent risk factor for poststroke dysphagia (OR 1.34); on the other hand, NIHSS also predicted the persistence of dysphagia at 14 days from symptoms onset (OR 1.5). By means of receiver operating characteristics (ROC) curves obtained by multivariable logistic model, we evaluated the predictivity of each significant variable using the area under the curve (AUC) value. The NIHSS score of 11.5 was found to be the best predictive value of persistent dysphagia; we also found an AUC of 0.898, which means that stroke severity at admission allowed to correctly classifying about 90 of 100 dysphagic patients in a persistent or non-persistent pattern. Hence, we suggested an NIHSS ≥12 as cut-off value in order to predict, upon admission, those patients who will probably remain dysphagic after 14 days follow-up. In comparison to Henke, study of our ROC analysis had a lower sensitivity 72.4 vs. 77% but a far better specificity 90.1 vs. 77%.

In this view, it could be interesting to combine the results of the two NIHSS cut-off: low probability of dysphagia if lower than 4.5, high probability of persistent dysphagia if more than 11.5, and a gray area from 4.5 to 11.5. It could be then speculated that this gray area probably represents those patients with a transient dysphagia (about an half of acute dysphagic patients) (3), that we found to have a lower NIHSS in respect to patients with persistent dysphagia (both in the acute phase and after 14 days), and that will probably recovery from dysphagia in the sub-acute phase of stroke.

## Author Contributions

MT: manuscript preparation; AV: references suggestion and critical review; and VP: manuscript preparation and critical review.

## Conflict of Interest Statement

The authors declare that the research was conducted in the absence of any commercial or financial relationships that could be construed as a potential conflict of interest.
